# What a weight loss programme should contain if people with obesity were asked - a qualitative analysis within the DO:IT study

**DOI:** 10.1186/s12889-020-09850-8

**Published:** 2021-01-06

**Authors:** Christina Jessen-Winge, Pia Maria Ilvig, Heather Fritz, Carl J. Brandt, Kim Lee, Jeanette Reffstrup Christensen

**Affiliations:** 1 Department of Midwifery, Physiotherapy, Occupational Therapy and Psycomotore therapy, University College Copenhagen, Sigurdsgade 26, 2200 Copenhagen, Denmark; 2 Department of Public Health, University of Southern Denmark, JB Winsløwsvej 9A, 5000 Odense C, Denmark; 3 Department of Health Care sciences, Occupational Therapy Program, Wayne State University, 259 Mack Ave, Detroit, MI 48201 USA; 4 Department of Occupational Therapy, University College South, Degnevej 16, 6705 Esbjerg, Denmark

**Keywords:** Occupational therapy, Overweight, Client-centered, Habits, Everyday life, Meaningfulness, Danish obesity intervention trial

## Abstract

**Background:**

Currently 1.9 billion adults worldwide are estimated to be overweight or obese. In Denmark the municipalities hold the responsibility to deliver weight loss programmes to overweight and obese citizens. There is a tendency to assume that weight loss programmes that show positive effects in specialized hospital settings are directly transferrable to municipal settings. However, municipality-based weight loss programmes have not produced clinically significant reductions in body weight. One reason for this may be that much research evidence regarding obesity programming neglects the perspectives of people with obesity. The first step in developing a weight loss programme designed for municipal settings is to understand what people with obesity want and need from a programme. The aim of this study was to examine what people with obesity find important in a weight loss programme for weight loss and weight maintenance.

**Methods:**

We used a qualitative, explorative, descriptive design with individual interviews. We included men and women age 17 and older with a BMI ≥ 25 kg/m2. Participants were recruited from the wait lists of 13 municipality programmes and through Facebook posts. Data were analyzed using content analysis.

**Results:**

Thirty-four participants with overweight or obesity were individually interviewed (ages between 19 and 74). Findings suggest that weight loss programmes should; a) support participants in structuring days; b) consider the use of replacement activities to reduce cognitive and emotional burden; c) aide individuals to increase self-efficacy and; d) include family and friends as well as health professionals and peers in the weight loss process. Diet and exercise, while important, should be balanced with other meaningful activities in everyday life.

**Conclusion:**

Participants in this study wished to balance weight loss related activities with overall everyday life as well as finding the believe in their ability to lose weight in social relations.

## Background

Currently 1.9 billion adults worldwide are estimated to be overweight (Body Mass Index (BMI) ≥ 25 kg/m2) or obese (BMI ≥ 30 kg/m2) with rates projected to continue to increase [1]. Though estimates vary, in Denmark 40–55% of the population is overweight or obese [2] with the prevalence being higher among males than females [3].

Overweight and obesity increase the risk of developing type 2 diabetes, heart disease, and stroke [4]. Obesity is also associated with social discrimination [4] and difficulties participating in daily activities like shopping and dancing [5, 6] which negatively influences quality of life [7]. The Danish Health Authority recommends formalized weight loss programming for treatment of overweight and obesity [2]. The Danish health care system is free for all and programmes are paid for by the tax regulating system. The health care system is divided in a) a region responsibility, where patients are treated in specialized units in hospitals (often people with BMI + 40 combined with other lifestyle related diseases), and b) a municipal responsibility for health promotion and prevention services for citizens not yet sick, but in risk of a lifestyle disease (often people with a BMI between 25 and 40) [8]. The municipalities include municipal health centers and General Practice (GP) clinics. The health centers offer weight loss programmes and GP primarily refer citizens to the programmes [8].

Dietary changes in combination with physical activity remain the frontline treatment in weight loss programmes [9–12]. Decades of research demonstrate, however, that individuals face a myriad of challenges when changing those behaviors. As such, most programmes provide educational content as well as multiple behavior change strategies such as goal setting, action planning, or self-monitoring [13–15]. Though some weight loss programmes have resulted in significant weight reductions [10, 12], the most successful programmes are often lengthy, dose intensive, and require specialized interventionists [10]. While there has been a tendency to assume the results of inpatient programmes are transferrable to municipal settings, data suggest that this is not the case [16].

There are several plausible explanations for the reported lack of efficacy of municipal weight loss programmes. Municipal programmes include education about diet, exercise and different forms of cognitive therapy, but those components are not standardized, which contributes to a high degree of heterogeneity in terms of programme content, delivery structure and dose [17]. The lack of standardization could be due to the fact that professionals from a variety of disciplines develop and conduct programmes across the different municipalities [17]. Another explanation could be that Danish people who receive obesity treatment through municipal programmes have different needs and expectations for weight loss programmes than people who are referred to hospitals [18].

Since the municipalities already offer programmes that include content on diet and exercise [17], it is possible that another challenge with the programmes stem from a lack of understanding about ‘how’ the municipalities should implement the content and best support the weight loss and weight maintenance process [19]. Building an effective municipal-based weight loss programme requires an understanding of existing research evidence as well as the perspectives of people with obesity [20]. Our goal is to develop a municipal level, evidence-based programme to reduce overweight and obesity and enhance the quality of weight loss programmes. The programme is called Danish Obesity Intervention Trial (DO:IT). The first step in this process is to understand what people with obesity want and need from a municipal-level weight loss programme [21]. Asking citizens about their needs and expectations has been shown to increase the efficacy of clinical practices [22, 23]. Qualitative studies of people who have attended weight loss programmes in general show that individuals prefer to build trustful and supportive relationships with providers and peers and focus on motivation before being pushed to change their exercise and dietary behaviors [24–26]. Those studies are based on individuals’ who have completed an existing programme and the weight loss programme experience therefore influences the results. Consequently, it is relevant to understand the perceptions of those with overweight or obesity who have never attended a weight loss programme as to avoid preconceived notions of what a programme should include. Also, most people with weight problems have tried to lose weight, though outside of formalized programmes, and as such they might have some important ideas about what could work. Creating a programme based on their perspectives could lead to greater programme success [22, 23]. The aim with the present study was to understand what people with obesity who have not sought out municipal weight loss services would want and need in a programme.

## Methods

We chose to use a phenomenological and hermeneutical perspective inspired from the theory of Paul Ricoeur as the methodology is most appropriate for describing and interpreting the central nature of human experience [27].

Ethical approval for this study was not necessary according to ‘The Regional Committees on Health Ethics for Southern Denmark’.

### Recruitment and participants

Participants were males and females age 17 and older with a BMI ≥ 25 kg/m2. We included participants at two levels of readiness for addressing weight loss [28]; those who had taken a step to lose weight by signing up for a municipal programme (preparation) and those who were contemplating change. Participants who had completed a weight loss programme in the context of municipality were excluded. The participants were thus recruited in two different ways.

The participants who had taken a step to lose weight were recruited from wait lists for weight loss interventions offered by 13 different municipalities in Denmark (Aarhus, Copenhagen, Esbjerg, Holsterbro, Odense, Randers, Slagelse, Skive, Svendborg, Syddjurs, Varde, Vejle and Vordingborg). Health professionals from the municipalities aided the research team in participant recruitment by phoning residents on the programme wait lists and making them aware of the study. Before contacting participants, we sent health professionals a description of the project to use in the recruiting process. Participants wanting to join the project gave verbal consent to health professional to have their contact information forwarded to the research team.

Participants who were contemplating weight loss, but who had not taken steps towards signing up for a program were recruited through a Facebook post. The post had a short description of the project including inclusion criteria. Three of the authors (CJW, PMI, JRC) posted the invitation with contact information on their Facebook pages. The post was thereafter shared 52 times. Participants who contacted the study team received a detailed description of the project both written and verbally. Participants did not receive any compensation for participating in the interviews.

### Data collection

We developed and obtained data from a semi-structured interview guide following the procedures described by Kvale and Brinkmann [29]. The number of interviews was directed by data saturation. The interview guide was divided into three sections. The first section contained questions about daily life starting with the question ‘Please describe a normal day’. The second section had questions about experiences with earlier weight loss attempts. The third section probed the participant’s wishes for the content, form and dose of a weight loss programme (see Table 1 for interview guide).

**Table 1:** Interviewguide

Interview questions	
*Daily life and overweight/obesity*	
- Please describe a normal day/weekend	
- How do you experience your daily life?	
- What is most important to you during the day/week?	
- Do you feel prevented in doing things you find important because of your overweight/obesity?	
- How do you experience time in your daily life related to changing lifestyle?	
- How would you plan your daily life to change lifestyle? Is there anything you would do more/less?	
*Experiences with weight loss attempts*	
- Please tell me about your experiences with weight loss attempt(s)? What did you do/not do to maintain the weight loss?	
- How do you experience not having succeed with keeping the weight loss?	
- Do you believe in succeeding with a weight loss process?	
- What was/is most important for your wish to lose weight? (social factors/fear of getting sick)?	
- Imagen you had succeed in a weight loss and kept the weight loss what do you believe would have supported this success?	
*Concrete wishes for the content of a weight loss program*	
- Based on your experiences with weight loss what would you expect for a weight loss program?	
- Have should the program be structured for you to participate? (what goals, content, group/individual, how long, where?).	
- Who or what could support you in a weight loss program? Both before, during and after?	
- How would you expect to be supported?	
- Have you ever rejected a weight loss program offered from the GP or others? Why?	
- Any last comments?	

The interview guide was pilot tested with two participants. Those tests led the research team to reorder the questions to promote a better flow, but the questions remained the same. The two pilot interviews were included in the dataset. Signed informed consent was obtained from every participant prior to any data collection. The interviews were conducted by the first author (a PhD student) and three female master students in Occupational Therapy. The first author is an experienced interviewer. Each student practiced the interview and was evaluated for competency by the first author prior to conducting interviews on their own.

The data were collected between January 2018 and March 2018. The interviews were conducted in Danish and they lasted between 30 and 65 min and were conducted at a place and time convenient to the participant. Most of the interviews were conducted in participants’ homes, but nine occurred in a University classroom. Interviews were audio recorded and subsequently transcribed by the same interviewer who had conducted the interview.

### Analysis

Transcripts were analyzed using qualitative content analysis [30]. The analysis focused on the manifest and latent content in order to capture the descriptive and the interpretative understanding of the text [27, 30]. The analysis was carried out as a four step process [31]. In the first step, two of the authors (CJW, KL) read the text twice to get an overall impression of the data. Second, the content related to the study aim was identified and divided into meaning units. In the third step, the meaning units were condensed and labeled with a code. All codes were then arranged into 12 subcategories and further into six categories, to give a comprehensive understanding of all the interviews [31]. The subcategories and categories were discussed between the two authors (CJW, KL) who reached agreement. The content of each category was discussed, refined and modified during an iterative process until the themes seemed to fit the data in the best possible way. Finally, we engaged in peer debriefing with the other authors and the findings were discussed. When different viewpoints arose, they were discussed and resolved through consensus. A native English scientific writer translated the quotations in the present study from Danish to English.

## Results

We interviewed 36 participants, at which point data saturation was reached (Table 2). Two participants were excluded because they had participated in a municipality weight loss programme. Twelve of the participants were males and 22 were females. Participants represented all regions in Denmark as well as rural and urban areas. All the participants had a desire to lose weight. Sixteen participants were recruited from the waiting list in the municipalities, 18 contacted the researchers because of the Facebook post.

**Table 2:** Participants included in the study

= 34	Frequency (%)
Gender:	
Female	22 (65%)
Male	12 (35%)
Age:	
19–30	8 (23%)
31–50	7 (21%)
51–70	17 (50%)
70 +	2 (6%)
Profession:	
Student	8 (23%)
Working	10 (20%)
Unemployed	3 (9%)
Pensionist	13 (38%)

Three themes resulted from the data analysis *1. Creating a structure for success, 2. Needing supports to make up for gaps in willpower, 3. Changing to doing something with positive meaning.*

### Creating a structure for success

When the participants talked about the opportunities for weight loss, they explained that it had to be ‘one thing at a time’. It was all about habits and they experienced that their days were filled up with habits related to their weight. P6 said:

*“I think it is about habits and there are so many. When I look at my daily life there are so many habits I need to change to reach the goal and I think it is not realistic to do it all at once.”* (Woman, 29 years old).

She further provided an example when explaining that the changes should start with breakfast and when a healthy breakfast was integrated in her daily life, she could then move on to change the habits she had related to lunch. P33, a 77-year-old women wanted to take ‘one day at a time’. Start with doing exercise 1 day a week, then 2 days and so on to develop the habit of doing exercise. This understanding of changing one habit at a time was based on participants’ experiences with dieting. Almost all the participants had experienced a diet-related eight loss failure. Specifically, while they had experienced losing weight, they also experienced gaining double the weight lost back. This was illustrated by P15:

*“I lost 20 kilos in three months, it was a really bad idea. I did not eat, I just drank coffee and smoked cigarettes. I starved ( … … …*) *and when I started eating my body just craved for more food and then I started getting really fat and gained 40 kilos.”* (Woman, 32 years old).

Participants noted that dieting simply resulted in an unhealthy “elevator-weight” and a feeling of failure. P5, a 29-year old woman, described it as a “smack in the face”. Diets were also regarded as boring and monotonous. There were so many restrictions. P2 felt like living in a jail:

*“Yes you only live a little. It is just the same. Get up, eat the same every day, exercise. It is just the same every day. You live in a jail. It feels that way anyway. Off course, I’ve seen results but it is no fun for a longer period.”* (Man, 27 years old).

Despite these statements, most of the participants talked about a desire to learn to be more structured and find the energy to persevere with weight loss. For some, staying engaged in meaningful activities reduced the willpower that it took to stick to healthy behaviors, as stated by P9:

*“It is better if you have something to do, so you can divert the attention from looking at the clock and see now it is 11 o’clock where I usually do this and that. But if you are on to something else at 11 o’clock, then you don’t think about eating.”* (Man, 49 years old).

The participants described the use of “replacement-activities” from which they could learn to do something new and through that, change their unhealthy habits. P29 a 57-year old woman gave an example with her habit of sitting on the sofa many hours during the day. This habit was combined with eating unhealthy snacks. He explained that if the sofa-habit were to change a change of the snack-habit would follow.

### Needing supports to make up for gaps in willpower

Participants desired weight loss and hoped that they would someday be able to lose weight. They also spoke about the current and future consequences that the inability to lose weight had on their lives. Many participants expressed fears of getting sick or not being able to see their children grow up. They also spoke about clothes that they used to wear when they were slimmer and that they still kept it in their closets. Their prior experiences of not being able to maintain lost weight fed their fears about the future and negatively influenced their self-efficacy. They talked about themselves as being lazy, having a weak character and having no self-regulation. They blamed themselves for the failures. P2 explained:

*“Yes I lost weight, I exercised but when I stopped I just couldn’t any more. If I did not eat what was on the diet plan I got so unhappy. I punished myself by saying: ‘you can’t do shit’ and all that stuff. And then I just gained the weight again.”* (Man, 27 years old).

Furthermore, the feeling of not being able to control their weight influenced their feelings about their appearance. Participants shared that they got sad when they looked in the mirror and expressed being disappointed by their looks and worried about how they looked to others’, especially in public. P4 said:

*“Oh, but it is not good when you get a bad self-image, you are a really fat girl and you are ugly and look at your breasts - they hang down at your stomach and your stomach is so big.”(…*.) *“Frankly, I am so fat by now that I don’t want to go to the swim and have people seeing me in a bikini.”* (Woman, 42 years old).

The combination of feeling as though one had no willpower to persevere towards weight loss efforts and feeling ‘fat’ resulted in a negative feedback loop where the more despondent participants became over their weight, the less willpower they had to change the situation. P28 explained that it was simply easier to find excuses for not changing.

*“I am good at those bad excuses even without knowing what is expected of me or knowing what I could win from doing it.”* (Woman, 70 years old).

The psychological toll that obesity took on participants was also reflected in their desire for more support. They expressed that they did not think they could succeed on their own and provided examples of the types of supports they felt would be most useful. Specifically, participants noted that supports should vary and come from different networks including from families and friends, from others with obesity and from health professionals. While participants agreed that they needed support, they differed in what type of support would work for them. For example, some participants stated that they needed criticism from friends and family, such as hearing that they were “too fat” to get motivated for weight loss. For P10, however, this type of criticism made him want to start eating even more.

“*… But it does not help … it will just mean that I go down to the city to buy candy and soda, ice cream and cake …*.” (Man, 57 years old).

This participant further explained that if somebody supported him in losing weight it should be somebody he respected, such as a girlfriend or a close friend.

When talking about staying committed to their weight loss efforts, the participants also noted that they needed more than verbal support. They needed friends and family to ‘do’ it with them, to agree to eat well and exercise together. P10 explained it like this:

*IP: “Yes ( …) that is why I often criticize my wife (laughing) because she does not want to go with me.”*

*I: “It would be easier if you were doing it together?”*

*IP: “Yes, I like running, it is so difficult for me to exercise without her.”* (Man, 57 years old).

Being together with peers with obesity was also of great importance for many participants. They wanted to feel like a community and spend time with others in the same situation and exchange experiences, not just about weight and weight loss, but about everyday life as well. P11 explained:

*“It doesn’t have to be about weight loss all of it, we could discuss how people integrate and do stuff that could be integrated in the everyday life as long as we do it together.”* (Man, 53 years old).

Besides talking and listing to others, the opportunity to exercise with peers with obesity was of great importance. P32 explained that she hated running in the city because she felt that everybody saw how fat she was and how badly she was running:

*“… .but doing it in a community where we felt as ‘US’ and none of us are used to running, would be much better.”* (Woman, 65 years old).

Regarding health professionals, participants wanted professionals to support them with knowledge and provide compliments and encouragement through the entire weight loss process. P5 described:

*“It is like with alcoholics anonymous, we need the support to be available all the time.”* (Woman, 29 years old).

### Changing to doing something with positive meaning

When the participants were asked directly to discuss the content that should be included in a weight loss programme most of the participants mentioned the importance of diet and exercise. Participants did not negate the need for knowledge about diet and physical activity, or the need to change their diet or activity levels. Participants emphasized, however, they wanted to have fun during the weight loss process. Further, they wanted the strategies they were taught from weight loss programmes to be applicable to their life circumstances. They had experienced that if this was not the case their efforts would not be successful.

Diet and exercise already had an enormous place in the participants’ lives. Many of the participants were already doing exercise, most of them in a fitness center where they described going many times during a week. Even when they were not physically exercising. P4 thought about it all the time: *“Yes, I run all the time … .inside my head.”* (Woman, 42 years old).

Among participants in our sample, exercise was described as an organized sport or something they had to do. Exercise was never considered an enjoyable activity. Most participants discussed how they got it done on the way home from work or school, so that they could come home to relax and do the things they enjoyed. P15 said:

*“So I can, it is all about when it is around 2 o’clock or something like that, then, oohh, then it is like … should I go home to sleep or lay on the sofa or should I get it over with. It is the energy deciding it.”* (Woman, 32 years old).

Many of the participants talked about finding pleasure in many other activities such as reading a book or spending time with friend or families. P4, a woman at 42 years old, said: *“exercise is good, but the sofa is better.”* P20 gave an example from when she exercised regularly, as she was aiming to run five kilometers without a break. And for *“every step on the way I moved further and further away from the things I liked doing.”* (Woman 29 years old).

Furthermore, to exercise was to be continually confronted with one’s obesity. P30 a 68-year old woman explained that she got “obesity-exhausted” when doing exercise because she felt that everybody was looking at her, reminding her of his weight.

When it came to diet, participants described how they differentiated food from ‘diet’. Food was something fun to make and to share with others. P25 stated.

*“Yes, I know where the problem is but we like eating cheese and bread and in our house, beer does not get too old – if you know what I mean.”* (Man, 66 years old).

Food could be enjoyed. Diet, however, was much more sterile and prescriptive. It was about being healthy versus having enjoyment. One participant described it as going from a fun thing (making and eating food) to a healthy thing (doing exercise). Thus, exercise competed or took away from other desired everyday activities and having to constantly think about what one was eating changed the meaning of food and food-related activities.

## Discussion

The aim of the present study was to understand what people with obesity find important in a weight loss programme without having ever participated in one. Our data suggest that participants desired a structured weight loss approach, that they needed varying levels of social support, and that they desired to change habits in a way that was fun and that did not devalue their existing meaningful activities. Participants’ perspectives support existing evidence regarding the desired components of weight loss programmes, while also suggesting new ways that the municipalities could integrate the desires and needs of people with overweight and obesity in the municipal weight loss programmes.

### Creating a structure for success

Participants’ desired to structure their weight loss efforts by mastering one thing at a time. At the same time, participants did not want a structure that dominated their lives, but rather one that provided a just-right amount of support and scaffolding for weight loss success. This sentiment is supported by prior research. For example, Gallagher et al. (2012) found that participants in a weight loss programme achieved greater weight loss when they integrated weight loss-related activities into a routine and focused on only doing one thing at a time, often beginning with exercising [32]. While most weight loss programmes discuss the importance of goal setting with participants, they may not be focusing on “one small thing at a time” [33]. Structuring weight loss programmes to allow participants to focus on and succeed with one small change at a time may also increase self-efficacy, which has been consistently linked to positive behavior changes [34].

In addition, by focusing on small, sequential changes programmes could include training on habit formation [35]. Habits are generally defined as behaviors that operate below conscious awareness and that are triggered by environmental cues. All behavior change requires modifying some habits [36]. One technique, among others, in developing new healthy habits, is using implementation intentions [36]. Implementation intentions are a form of ‘if-then goal setting’ that has been linked to successful behavior change. For example, if participants are hungry and it’s not yet time to eat, they will then do a crossword puzzle. Since our findings suggest that participants are already using “replacement-activities,” the use of implementation intentions could be a way for weight loss programmes to assist participants in identifying and implementing even more replacement activities. A recent qualitative study by Cleo et al. suggested that participants found value in using replacement activities to change habits and that such approaches contributed to their successful weight loss maintenance because the habits were implemented in their daily life [33].

### Needing supports to make up for gaps in willpower

Participants reported that they sometimes felt lazy and as if they had a weak character, and women in particular referred to themselves as being ugly. This way of describing the self may reflect the popular discourse of ‘healthism’, which suggests that health can be achieved through individual effort and discipline. From this point of view, people with obesity are characterized as lazy, self-indulgent and greedy [37]. When this discourse is combined with the experiences of not being able to maintain weight loss, it is not surprising that participants felt unable to lose weight on their own. Existing research has noted the importance of social support to general health and wellbeing [38, 39]. Specifically, the sense of being included in social groups increases the feeling of personal autonomy and thereby increase the belief that one is capable of producing behaviours that lead to a specific desired effect [40]. Our participants specified that social support was particularly important to overcoming gaps in willpower that stemmed from negative feelings about one’s self. As such, municipalities should consider how best to integrate multiple levels of social support into weight loss programming. Health professionals and peer support has especially been shown to be important to foster commitment to the weight loss process [21]. Though participants in our study wished to include health professionals and peers in a weight loss programme, they also emphasized the need to include individuals in their closest social networks (e.g., significant others, family, or friends) especially by doing activities together with them. In our study this desire was expressed by both men and women. Though prior research is mixed regarding who benefits the most from social support according to gender [40], it is known that close social relationships play a role in the development of overweight and obesity for both genders [41]. Therefore, it is plausible that participants’ desires to include close social relationships in weight loss programmes could have the reverse effect and aid in weight loss and weight maintenance.

### Changing to doing with positive meaning

Combining diet and exercise in a weight loss process is a common recommendation and therefore, it is not surprisingly that participants mentioned this as a necessary content of a weight loss programme. Our participants, however, specified the different meanings of diet and exercise. Preparing and eating food were meaningful in a positive sense while diets and exercise had a negative meaning. Those results are supported by a qualitative study from Thomas et al., in which participants attributed negative meanings to exercise due to the difficulties of engaging in exercise because of their bodyweight. The participants perceived that exercise was hard work whereas diets were seen as a ‘quick fix’, but without a sustaining effect [42].

When the participants in our study discussed dietary changes and exercise, they acknowledged the value of those behaviors for weight loss. However, it seems that the understanding of diet and exercise were linked very narrowly to sports and calories and were not always congruent with their personal values and norms. They also noted that such pursuits were physically and emotionally taxing and took time away from activities they found meaningful. Engaging in activities that are not congruent with one’s own values and norms could result in negative consequences and activity imbalances [43]*.* Activity imbalance and balance is a way to understand how all the things that people do during the day and the entire life affect meaningfulness and wellbeing [44]. To thrive, humans typically need to participate in a variety of activities with different meanings [45]. Thus, understanding how recommended weight loss activities such as dietary changes and exercise effect the well-being and activity balance in everyday life should be a part of a weight loss programme.

### Strength and limitation

We wanted to understand the wishes for at weight loss programme related to the municipalities. Even though there are both hospitals and private providers in Denmark offering treatments for people with obesity, the municipalities are the most important actor in the Danish health care system related to weight loss programmes. We chose to include only participants who did not attend a weight loss programme in either the municipality or the hospital setting. Including participants with those experiences could have led to different perspectives to the aim of understanding the wishes for a weight loss programme. However, the strengths of this study are the use of a purposeful, theoretically informed sampling strategy and the large sample diverse in age, gender and employment. Thus, our participants represent broad perspectives that are essential in the development of a weight loss programme directed to the municipalities.

Our study was limited by our decision to allow participants to address the authors if they wanted to be interviewed. We made this choice to overcome the challenge of stigmatization. This decision may have led to a lack of representation from people with lower social status, those who do not use Facebook, or people who are not used to talking about experiences from their everyday life. Therefore, the participants in the present study might not represent the experiences of all people with overweight or obesity. All interviewers were also occupational therapist. As occupational therapists are concerned with activities and meaning in everyday life, it is likely that the disciplinary orientation of the study team influenced how they framed and interpreted the data.

## Conclusion

Overall our finding suggests that municipal weight loss programmes should focus on everyday life to find balance in activities that are meaningful for the participants instead of focusing solely on weight loss related activities as diet and exercise. This process should be done in small steps. Further, the importance of social support is highlighted as well as the interconnection of self-belief, self-efficacy, and social relations.

Implication for future research and practice: Our findings complement the existing evidence with knowledge about supporting people with obesity to structure their days to find a meaningful balance between diet and exercise together with other meaningful activities in their everyday life. Future research should understand the effectiveness of a meaningful balance within everyday life in a weight loss program.

Further the generic understanding from this study could be further enhanced by different perspectives based on social status and BMI to improve the possibility to develop a program to more specific groups.

## Data Availability

The datasets used and analyzed during the current study are available from the corresponding author on reasonable request.

## Abbreviations

DO:IT: Danish Obesity Intervention Trial
BMI: Body Mass Index
I: Interviewer
IP: Interview participant
